# The Association of Intravitreal Injections of Different Anti-Vascular Endothelial Growth Factor with Systemic Outcomes in Diabetic Patients

**DOI:** 10.3390/jpm13030544

**Published:** 2023-03-18

**Authors:** Eugene Yu-Chuan Kang, Tzu-Yi Lin, Sunir J. Garg, Nan-Kai Wang, Lee-Jen Chen, Pei-Wei Huang, Ming-Jen Chan, Kuan-Jen Chen, Wei-Chi Wu, Chi-Chun Lai, Yih-Shiou Hwang

**Affiliations:** 1 Department of Ophthalmology, Chang Gung Memorial Hospital, Linkou Medical Center, Taoyuan 333, Taiwan; 2 College of Medicine, Chang Gung University, Taoyuan 333, Taiwan; 3 Graduate Institute of Clinical Medical Sciences, Chang Gung University, Taoyuan 333, Taiwan; 4 Department of Education, Chang Gung Memorial Hospital, Linkou Medical Center, Taoyuan 333, Taiwan; 5 MidAtlantic Retina, The Retina Service of Wills Eye Hospital, Thomas Jefferson University, Philadelphia, PA 19107, USA; 6 Department of Ophthalmology, Edward S. Harkness Eye Institute, Columbia University Medical Center, New York, NY 10032, USA; 7 Department of Ophthalmology, Mackay Memorial Hospital, Taipei 104, Taiwan; 8 Department of Oncology, Chang Gung Memorial Hospital, Linkou Medical Center, Taoyuan 333, Taiwan; 9 Kidney Research Center, Department of Nephrology, Chang Gung Memorial Hospital, Linkou Medical Center, Taoyuan 333, Taiwan; 10 Department of Ophthalmology, Keelung Chang Gung Memorial Hospital, Keelung 204, Taiwan; 11 Department of Ophthalmology, Jen-Ai Hospital Dali Branch, Taichung 412, Taiwan; 12 Department of Ophthalmology, Xiamen Chang Gung Memorial Hospital, Xiamen 361000, China

**Keywords:** intravitreal injection, anti-vascular endothelial growth factor, systemic outcomes

## Abstract

This retrospective cohort study aimed to assess the systemic effects of three commonly available anti-vascular endothelial growth factor intravitreal injections in patients with diabetes, using data taken from a multi-institutional database in Taiwan. Patient data were sourced from the multi-institutional Chang Gung Research Database. Participants were divided into groups based on treatment with bevacizumab, ranibizumab, or aflibercept. Baseline characteristics were matched among the groups by the inverse probability of treatment weighting. The incidence rate of outcome events was calculated as the number of events divided by 100 person-years of follow-up. The cumulative incidence function was used to estimate the incidence rate of the outcome events among groups. The incidence of ischemic stroke was higher in the ranibizumab group than the bevacizumab and aflibercept groups (1.65, 0.92, and 0.61 per 100 person-years, respectively). The incidence of major adverse lower-limb events was higher in the bevacizumab group (2.95), followed by ranibizumab (2.00) and aflibercept (0.74). Major bleeding was relatively higher in bevacizumab (12.1) compared to ranibizumab (4.3) and aflibercept (3.8). All-cause death was higher for both bevacizumab (3.26) and aflibercept (2.61) when compared to ranibizumab (0.55), and all-cause admission was found to be highest with bevacizumab (58.6), followed by aflibercept (30.2), and ranibizumab (27.6). The bevacizumab group demonstrated a greater decrease in glycated hemoglobin compared to the baseline level (−0.33%). However, a few differences in the clinical condition between the groups were still observed after matching. In conclusion, this study suggests that different anti-vascular endothelial growth factor agents may be associated with various and differing systemic adverse events. The differences might also be attributed to differences in patient characteristics and clinical status.

## 1. Introduction

Diabetes mellitus is a widespread chronic disease, and its incidence continues to rise [1]. Beyond imposing a heavy burden on health infrastructure, it also leads to life-threatening macrovascular and microvascular complications [2]. In addition, a persistent hyperglycemic state can induce inflammation and angiogenesis in the retina [3]. Ocular disability resulting from diabetic retinopathy (DR) has a significant impact on patients’ daily lives. Diabetic macular edema (DME), proliferative diabetic retinopathy (PDR), and diabetic retinopathy (DR) in general are leading causes of vision loss in patients older than 40 years of age [4]. While retinal laser coagulation and the intravitreal injection (IVI) of corticosteroids still play essential roles in treating DME [5,6], intravitreal injection of anti-vascular endothelial growth factor (VEGF) agents has transformed the standard of care for retinal diseases [7].

VEGF increases capillary permeability and causes a breakdown of the blood–retinal barrier [8]. The resulting leakage of fluid into the retina can significantly affect vision. Anti-VEGF agents, including bevacizumab, ranibizumab, and aflibercept, have been extensively used for numerous retinovascular diseases. While ranibizumab and aflibercept are indicated only for ocular disease, the initial application of bevacizumab was as intravenous chemotherapy for colorectal, breast, and lung cancers. Intravenous bevacizumab has been associated with systemic adverse events, including hypertension, proteinuria, myocardial infarction, and stroke [9,10,11]. Furthermore, an elevated mortality rate has also been noted in patients treated with a combination of bevacizumab and chemotherapy [12]. Although only 0.05 mL of intravitreal anti-VEGF medications are injected in an eye, anti-VEGF agents still enter the systemic circulation [13,14]. Unilateral therapy has been shown to decrease VEGF levels in serum and regress the neovascularization of contralateral eyes [15]. Consequently, there has been interest in assessing the potential systemic effects noted after the IVI of anti-VEGF agents [16].

In several clinical trials, the various anti-VEGF agents demonstrated a low incidence of adverse events [17,18,19]. However, given the systemic comorbidities associated with diabetes and the relatively uncommon nature of these events, the registry studies are underpowered to assess systemic impact. Different inclusion and exclusion criteria used for the various studies can also impact the incidence of adverse events from anti-VEGF agents. In addition, findings derived from clinical trials may have limited applicability in populations that fall outside of the inclusion and exclusion criteria [20]. In contrast, real-world data provide robust evidence to investigate systemic adverse effects. Some studies have suggested that anti-VEGF agents increase thromboembolic events and associated death [21,22], whereas other studies have found anti-VEGFs to be safe and without an elevated risk of major cardiac adverse events [23,24]. Despite these conflicting reports, the available data are limited. This study aims to assess the systemic association of three commonly used anti-VEGF agents in patients with diabetes mellitus, using data taken from a multi-institutional database in Taiwan.

## 2. Materials and Methods

### 2.1. Data Source

This retrospective cohort study was conducted using the Chang Gung Research Database (CGRD), a multi-institutional electronic medical record database of 1.3 million patients across Taiwan. The database has de-identified clinical information, including clinical diagnosis, medication use, interventions, laboratory data, and operation notes, as well as other data obtained during routine clinical care. In addition, the CGRD contains information on self-pay items that were not covered by the Taiwan National Health Insurance program. The database was queried using International Classification of Diseases, Ninth Revision, Clinical Modification (ICD-9-CM) diagnostic codes before 2015 and both ICD-9-CM and ICD-10-CM after 2016. The detailed information on CGRD has been described in previously published studies [25,26]. All procedures adhered to the principle of the Declaration of Helsinki. Written informed consent was waived due to the de-identification of the data. This study has been approved by Chang Gung Medical Foundation Institutional Review Board (IRB No. 202200606B1).

### 2.2. Patient Inclusion

Patients receiving IVI with anti-VEGF between 1 January 2014 and 31 December 2019, were identified in the CGRD. The index date was defined as the day anti-VEGF treatment was initiated. The new user design was adopted to reduce the potential selection bias. Therefore, patients with previous IVI with any anti-VEGF agents before 2014 were excluded. Moreover, patients under the age of 20 years, patients without diabetes, those with no baseline glycated hemoglobin (HbA1c) data, patients with a history of having received IVI with steroids, and those with any preexisting malignancy were also excluded. Patients were then grouped according to the anti-VEGF treatments. In Taiwan, this includes bevacizumab, ranibizumab, and aflibercept.

### 2.3. Covariates

The covariates were demographics (sex, age, and body mass index (BMI)), the severity of DM, systemic comorbidities, Charlson’s Comorbidity Index (CCI) score, medication use, ocular history, and the number of outpatient visits to ophthalmology in the previous year prior to treatment. The severity of DM was assessed by the HbA1c, duration of diabetes, DM complications (diabetic neuropathy and diabetic foot ulcers), and the type of DM. Comorbidities included metabolic syndrome (hypertension and dyslipidemia), cardiovascular disorders (ischemic heart disease, ischemic stroke, heart failure hospitalization, myocardial infarction, and atrial fibrillation), and other diseases (chronic kidney disease, chronic obstructive pulmonary disease, and obstructive sleep apnea). The Charlson Comorbidity Index score was calculated to evaluate the burden of disease [27]. Medication usage catalogued at the index date included anti-platelets, anti-coagulants, statins, and fibrates. Prior ocular history included glaucoma, age-related macular degeneration, diabetic macular edema, retinal vascular occlusion, vitreous hemorrhage, myopic choroidal neovascularization, all-grade diabetic retinopathy, and whether an eye received retinal laser and/or vitrectomy for any indication.

### 2.4. Outcomes

Outcomes were clinical events and changes in the laboratory data. Clinical events included all-cause death, all-cause hospital admission, major adverse cardiac event (an one of myocardial infarction, ischemic stroke, and cardiovascular death), major adverse lower-limb event (MALE) outcomes (peripheral arterial disease, claudication, critical limb ischemia, percutaneous transluminal angioplasty, and amputation), composite thromboembolic events (myocardial infarction, ischemic stroke, transient ischemic attack, extremity thromboembolism, and systemic thromboembolism), and major bleeding requiring hospitalization. The date, place, and cause of death were identified using the Taiwan Death Registry, which is released by the Taiwan Ministry of Health and Welfare. The occurrence of the following events was assessed during hospitalization: myocardial infarction, ischemic stroke, percutaneous transluminal angioplasty, amputation, and transient ischemic attack. The occurrence of diseases was defined as having outpatient diagnoses from at least two visits or an inpatient diagnosis at least once. Patients were followed up until individual clinical events, the 180th day after the index date, the day of death, the day of a switch between the three study drugs, the last visit in the CGRD, or 31 December 2019, whichever came first.

Laboratory data were extracted at baseline and at the sixth month after the index date. Laboratory data of interest were the systolic blood pressure (SBP), diastolic blood pressure (DBP), HbA1C, low-density lipoprotein (LDL), estimated glomerular filtration rate (eGFR), and alanine aminotransferase (ALT). The change of laboratory data from baseline to the sixth month after the index date was analyzed.

### 2.5. Statistical Analysis

When comparing the risk of clinical events among multiple anti-VEGF agents (bevacizumab vs. ranibizumab vs. aflibercept), an additional adjustment cohort was created using an inverse probability of treatment weights (IPTW) based on propensity scores (PSs). As there were multiple treatment groups (>2 groups) in this study, we estimated the PSs using the generalized boosted model based on 50,000 regression trees [28]. The variables included in the PSs estimation are listed in Table 1. However, the number of injections during the 6 month follow-up was not included. The balance among the multiple anti-VEGF agents before and after IPTW was assessed using maximum absolute standardized differences (MASD) in which a value less than 0.1 indicated a negligible difference and a value larger than 0.2 indicated a substantial difference among the groups [28]. The covariates with a MASD value >0.1 (non-negligible difference) in the IPTW-adjusted cohort were further additionally adjusted in the subsequent multivariable analysis.

The incidence of clinical events was expressed using incidence density, which denoted the number of events per 100 person-years. The incidence of clinical events and the subsequent survival analyses were estimated in the IPTW-adjusted cohort. The risk of fatal outcomes (i.e., all-cause mortality) among the multiple anti-VEGF agents was compared using the Cox proportional hazard model. The incidence of other non-fatal time-to-event outcomes (i.e., major bleeding requiring hospitalization) among the multiple anti-VEGF agents was compared using Fine and Gray sub-distributional hazard model, which considered all-cause mortality a competing risk. The changes in laboratory data from baseline to the sixth month among the multiple anti-VEGF agents were compared using a linear mixed model in which the baseline value (intercept) and slope were set as random effects. A two-sided *p* value <0.05 was considered statistically significant. Statistical analyses were performed using SAS version 9.4 (SAS Institute, Cary, NC, USA).

## 3. Results

This section may be divided by subheadings. It should provide a concise and precise description of the experimental results, their interpretation, and the experimental conclusions that can be drawn.

### 3.1. Patient Enrollment

**Figure 1:**
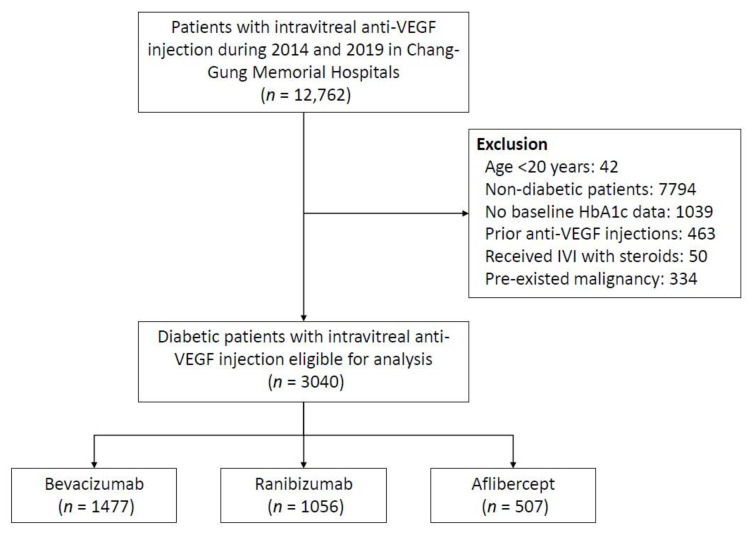
Flowchart of study cohort assembly. Anti-VEGF—anti-vascular endothelial growth factor; HbA1C—glycated hemoglobin; IVI—intravitreal injection.

A total of 12,762 patients receiving IVI with anti-VEGF were identified. Of these patients, 9722 were excluded, leaving 3040 diabetic patients receiving IVI with anti-VEGF (Figure 1). These patients received either IVI with bevacizumab (1477 patients), ranibizumab (1056 patients), or aflibercept (507 patients).

### 3.2. Baseline Characteristics

The baseline demographic and clinical data gathered prior to IPTW were compared according to different anti-VEGFs, and substantial differences (MASD > 0.2) were noted for several characteristics (Supplemental Table S1). A greater proportion of patients receiving bevacizumab also had a chronic kidney disease comorbidity (52.1%) when compared with patients treated with ranibizumab (39.3%) and aflibercept (34.5%). Regarding ocular history, a greater proportion of patients receiving aflibercept (47.1%) had a history of age-related macular degeneration compared to patients treated with bevacizumab (19.8%) and ranibizumab (24.1%). A greater proportion of patients receiving bevacizumab had a greater incidence of vitreous hemorrhage (30.7%) compared with patients treated with ranibizumab (15.7%) and aflibercept (11.4%). Fewer patients treated with aflibercept had a history of all-grade diabetic retinopathy (63.9%) when compared to patients receiving bevacizumab (71.6%) and ranibizumab (83.0%). Similarly, fewer patients treated with aflibercept had received retinal laser therapy (29.0%) compared to patients receiving bevacizumab (41.3%) and ranibizumab (46.9%).

The distribution of baseline characteristics among the three study groups was more balanced after IPTW adjustment (Table 1). However, there were still substantial differences (MASD > 0.2) for several variables, including chronic kidney disease, age-related macular degeneration, vitreous hemorrhage, all-grade diabetic retinopathy, and retinal laser. The prevalence of chronic kidney disease was higher in the bevacizumab group than the aflibercept group (47% vs. 35.7%). In the aflibercept group, the prevalence of age-related macular degeneration was the highest, while the prevalence of diabetic retinopathy and retinal laser was the lowest. The prevalence of vitreous hemorrhage was the highest in the bevacizumab group. The prevalence of any diabetic retinopathy was the highest in the ranibizumab group. The variables with non-negligible differences after IPTW (MASD > 0.1) will be further adjusted in the subsequent outcome analyses (either clinical events or laboratory outcomes).

### 3.3. Clinical Events

**Figure 2:**
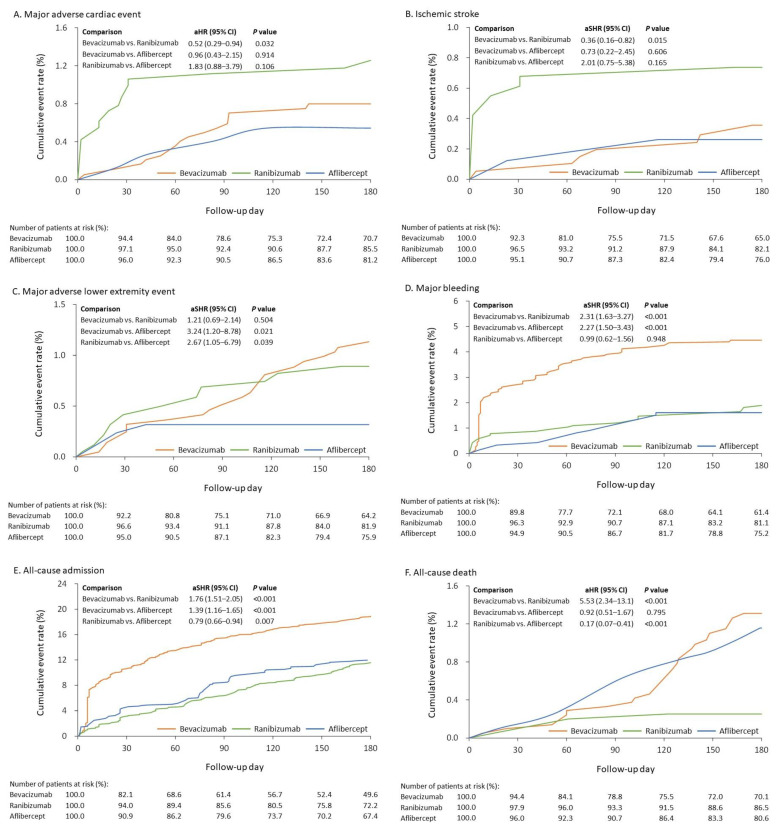
Cumulative event rate of (**A**) major adverse cardiac event, (**B**) ischemic stroke, (**C**) major adverse lower extremity event, (**D**) major bleeding, (**E**) all-cause admission, and (**F**) all-cause death for patients receiving different anti-vascular endothelial growth factor agents in the IPTW-adjusted cohort. aHR—adjusted hazard ratio; aSHR— adjusted sub-distribution hazard ratio; CI—confidence interval; IPTW—inverse probability of treatment weighting.

The results of comparing the risk of outcomes among different anti-VEGFs after IPTW adjustment are listed in Table 2. During the follow-up of major adverse cardiac events, the cumulative event rate was significantly lower in patients that received bevacizumab compared to ranibizumab (adjusted sub-distribution hazard ratio (aSHR) 0.52, 95% CI: 0.29–0.94) (Figure 2A). This result was mainly driven by ischemic stroke (Figure 2B). The risk of a MALE outcome was significantly higher in patients receiving either bevacizumab or ranibizumab compared to aflibercept (bevacizumab vs. aflibercept—aSHR: 3.24, 95% CI: 1.20–8.78; ranibizumab vs. aflibercept—aSHR: 2.67, 95% CI: 1.05–6.79) (Figure 2C). Incidences of major bleeding requiring hospitalization were higher in patients receiving bevacizumab than for the other two agents (Figure 2D). A statistically increased risk of all-cause death was observed in the bevacizumab and aflibercept groups when compared to the ranibizumab group (bevacizumab vs. ranibizumab—adjusted hazard ratio (aHR): 5.53, 95% confidence interval (CI): 2.34–13.08; ranibizumab vs. aflibercept—aHR: 0.17, 95% CI: 0.07–0.41) (Figure 2F). The risk of all-cause admission was significantly higher in patients receiving bevacizumab, followed by aflibercept and then ranibizumab (Figure 2E).

### 3.4. Laboratory Outcomes

**Figure 3:**
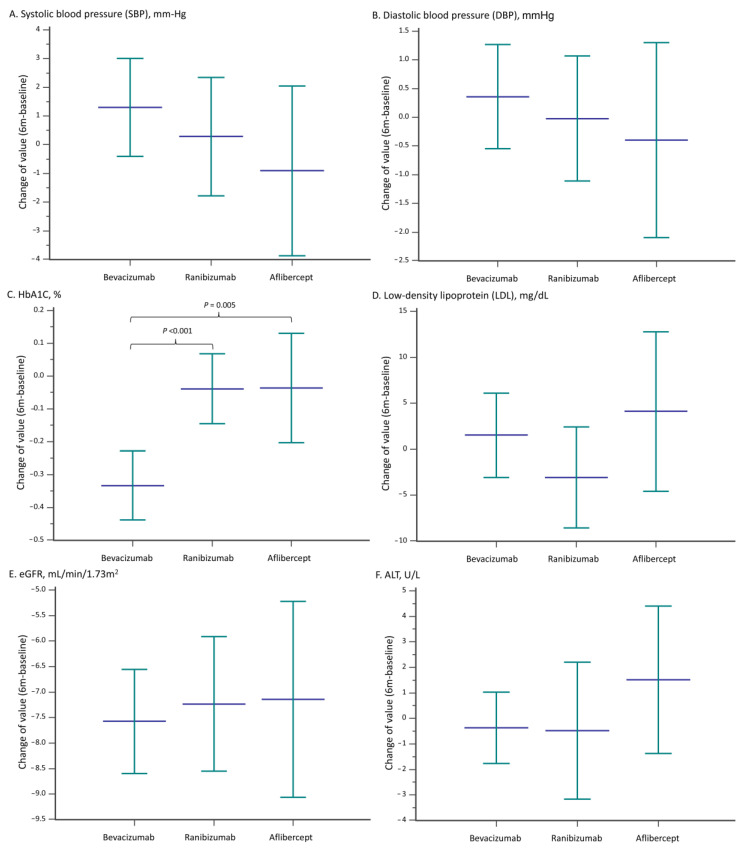
Changes from baseline to six months of (**A**) systolic blood pressure, (**B**) diastolic blood pressure, (**C**) HbA1C, (**D**) low-density lipoprotein, (**E**) eGFR, and (**F**) ALT for patients receiving different anti-vascular endothelial growth factor agents in the IPTW-adjusted cohort. ATL—alanine aminotransferase; eGFR—estimated Glomerular filtration rate; HbA1C—glycated hemoglobin; IPTW— inverse probability of treatment weighting.

Changes in baseline measurements after six months were also assessed (Figure 3). No statistically significant differences in the changes from baseline to the sixth month were observed for SBP, DBP, LDL, eGFR, and ALT among the three study groups. However, a significant drop in HbA1c was observed in patients receiving bevacizumab (mean ± standard deviation (SD): −0.33 ± 1.65%) than those receiving ranibizumab (−0.04 ± 1.38) or aflibercept (−0.04 ± 1.36) during the 6 month follow up (Figure 3C).

## 4. Discussion

Clinical trials have demonstrated that anti-VEGF treatment carries a low adverse event incidence. This is despite bevacizumab having been associated with adverse events and an elevated mortality rate when used as an intravenous chemotherapeutic. While an anti-VEGF is administered at doses far lower than those used in oncology, adverse events may still occur but go undetected, possibly because clinical trials are underpowered for these outcomes or are impacted by the eligibility criteria used in trial enrollment. The current literature on the association between adverse events and anti-VEGF treatment remains divided. This study aimed to assess the systemic adverse events of three anti-VEGF treatments in real-world diabetic patient data collected from a multi-institutional database in Taiwan.

In our study, the real-world patient data demonstrated differences in the incidence of systemic outcomes between different anti-VEGF therapies. A trend towards a significantly higher incidence of ischemic stroke was seen in patients receiving ranibizumab vs. those receiving bevacizumab or aflibercept. The MALE outcome was higher in patients receiving either bevacizumab or ranibizumab when compared to aflibercept. Major bleeding incidents requiring hospitalization were higher in patients receiving bevacizumab. All-cause admission was found to be significantly higher in patients receiving bevacizumab, followed by aflibercept and then ranibizumab. The time to event outcome analysis suggests that a significantly higher incidence of all-cause death was detected for both bevacizumab and aflibercept compared to ranibizumab. However, the composite thromboembolic events were comparable between the three groups.

Several differences were noted between the anti-VEGF groups when baseline demographic and clinical data were compared. Although an IPTW adjustment was used to balance intergroup differences, there were still substantial differences in several baseline characteristics, including the number of injections in 6 months, underlying chronic kidney disease, diagnosis of age-related macular degeneration, diagnosis of any-grade diabetic retinopathy, diagnosis of vitreous hemorrhage, and history of receiving retinal laser therapy (Supplemental Table S1). Regardless of the number of intravitreal injections, all-cause hospital admission was found to be the highest in patients receiving bevacizumab, followed by aflibercept and then ranibizumab. Major bleeding incidents requiring hospitalization were higher in patients receiving bevacizumab. The fitted cumulative incidence for all-cause admission and major bleeding increased soon after follow-up day 0 for bevacizumab compared to ranibizumab and aflibercept. This increase possibly suggests that the general condition of the patients receiving bevacizumab was worse than in the other groups, and this assumption could be affirmed by the higher rate of chronic kidney disease in the bevacizumab group compared to the others. In addition to the baseline difference, it has been previously noted that systemic exposure is higher for bevacizumab when compared to ranibizumab and aflibercept, and that systemic use of anti-VEGFs is associated with adverse effects such as exacerbating renal failure and proteinuria [29,30]. While the literature suggests that IVI with bevacizumab does not affect diabetic patients, those with preexisting renal dysfunction may be at risk of worsening albuminuria, and a recent case study reported worsening renal function in a diabetic patient treated with IVI bevacizumab [29,30,31,32].

Although we have used an IPTW adjustment to balance the age effect between groups, a higher proportion of age-related macular degeneration was found in the aflibercept group. In the baseline characteristic, patients receiving aflibercept treatment were also older than the patients in the bevacizumab and ranibizumab groups (Table 1) before the IPTW adjustment. These results may indicate a more degenerative health condition in the aflibercept group. This potential imbalance may also lead to higher mortality in the aflibercept group than ranibizumab group. Patients receiving ranibizumab had a higher rate of diabetic retinopathy, and retinal laser therapy was more commonly performed in this group. Diabetic retinopathy is the most common microvascular complication in diabetes [33]. An association between diabetic retinopathy and thromboembolic events has been reported [34]. This association could explain the higher incidence of MALE events and ischemic stroke in the ranibizumab group. Additionally, all-cause mortality is a competing risk of the cardiovascular outcomes in our study [35,36]. This may also explain the significantly lower incidence of major cardiac adverse events in the bevacizumab group, which had a higher incidence of all-cause mortality and a possibly worse baseline medical condition than the ranibizumab group. It has previously been demonstrated that anti-VEGFs can enter the systemic circulation, and systemic levels of VEGF were significantly lower in patients receiving bevacizumab and aflibercept compared to ranibizumab [14,15]. The reported lower VEGF levels, in combination with the current findings, suggest that the ability of different anti-VEGFs to enter systemic circulation might also contribute to systemic adverse events.

Although the anti-VEGF dose and the incidence of systemic adverse events in ophthalmology are much lower than those in oncology [37], and previous articles reported no significant association between IVI with anti-VEGF and all-cause admission and cardiovascular adverse events irrespective of diabetic status [38,39], the target study population in our study comprised vascular vulnerable patients (i.e., diabetic patients, 45% with chronic kidney disease) and may have a higher complication rate even under a low dose of anti-VEGF therapy. From the fitted cumulative incidence analysis, the systemic adverse events are usually observed within the 90 day follow-up (Figure 2) when the patients may receive the most frequent IVI with anti-VEGF (i.e., three monthly loading doses) [40]. In a systemic review with a meta-analysis, patients with diabetic macular edema were previously noted to have a higher risk of mortality that was slightly associated with an increasing number of anti-VEGF injections at twenty-four months [41]. Therefore, we would suggest monitoring not only ocular outcomes but also the general health conditions, such as blood pressure and cardiovascular signs, in patients who receive frequent anti-VEGF therapy, especially those with advanced age or diabetic complications.

Of the systemic lab parameters that were assessed, only HbA1C was demonstrated to be reduced in patients receiving bevacizumab (Figure 3). In Taiwan, the use of aflibercept and ranibizumab requires review by the national health insurance prior to administration [42,43]. For diabetic macular edema, patients must to fit the criteria, including an HbA1c level ≤10%, to receive the reimbursement for ranibizumab or aflibercept. Intravitreal bevacizumab injection was not covered by the national health insurance, and there were no criteria for using bevacizumab either. Higher HbA1c levels may disqualify patients from receiving aflibercept and ranibizumab, as these do not meet the NIH requirement (i.e., >10%). Additionally, the application for reimbursement may also be time-consuming. Given these hurdles, anti-VEGF therapy with bevacizumab may be chosen for the timely treatment of diabetic retinal complications. As visual disturbance is one of the first presentations of diabetes, and patients treated with bevacizumab often also receive a hypoglycemic agent with their diagnosis of diabetes [44]. Therefore, the HbA1c may be decreased after the bevacizumab treatment. Interestingly, no change in systolic or diastolic blood pressure was observed in this study, which corresponds to the research by Glassman et al. in which no treatment group differences in BP were detected between anti-VEGFs [31]. These results contrast with a previous study by Shah et al., which reported an association between elevated BP and anti-VEGF injections in diabetic patients with DR [45].

There were some limitations in this study. The retrospective design may have a possible selection bias present in the study population, posing a limitation. The baseline characteristics of the study groups could not be fully matched due to the wide variance in the factors within each group. While we employed IPTW to balance the variables, the different baseline conditions could still potentially impact the study results. Nevertheless, the findings of this study could still provide valuable information for real-world clinical practice regarding the use of anti- VEGF therapies for the treatment of eye diseases. Interim data may also miss if the patient did not have regular follow-up at the institutes. The patients visiting Chang Gung Memorial Hospitals, which are either secondary or tertiary medical institutes, and may have had more advanced disease conditions. In addition, patient characteristics available on the CGRD have previously differed from those found in Taiwan’s national database [25]. This study compared adverse events between anti-VEGFs and did not include other treatments such as dexamethasone implants. Furthermore, we did not compare patients with mixed anti-VEGF use. The identification of a control group without anti-VEGF therapy was challenging due to the use of a real-world, multi-institutional database, resulting in a lack of such a group in our study. To address this limitation, future research could explore alternative methods to provide a more comprehensive understanding of the effects of anti-VEGF therapy. It is worth noting that residual confounding may still be present despite the use of IPTW to balance the covariates. Further studies could explore alternative methods to minimize selection bias and address unmeasured confounding factors. Additionally, investigations on the long-term outcomes and safety of anti-VEGF treatments could provide a more comprehensive understanding of their clinical utility.

## 5. Conclusions

The main findings of this study suggest that different anti-VEGF agents may be associated with different systemic adverse events. While the baseline characteristics could not be fully matched across the study groups, the use of IPTW to balance the variables may reduce selection bias in this study. It is possible that these findings are related to different baseline characteristics or to the differences in anti-VEGF entering the systemic circulation. Despite potential limitations, the study results can still provide useful insights into the real-world effectiveness of anti-VEGF therapies in treating eye diseases. Monitoring the systemic adverse events is suggested in patients with advanced age or multiple diabetic complications.

## Figures and Tables

**Table 1:** Baseline demographics and clinical characteristics after inverse probability of treatment weighting.

Variable	Total (*n* = 3040)	Bevacizumab (*n* = 1477)	Ranibizumab (*n* = 1056)	Aflibercept (*n* = 507)	MASD
Male	57.4	57.6	59.1	54.6	0.09
Age, years	62.2 ± 11.8	61.4 ± 12.3	62.2 ± 11.6	63.2 ± 11.3	0.15
Age ≥ 65 years	41.1	38.1	42.0	44.0	0.12
Number of injections during 6 month follow up	3.1 ± 2.3	1.7 ± 1.1	4.2 ± 2.4	3.6 ± 2.2	0.90
BMI, kg/m^2^ *	25.8 ± 4.1	25.8 ± 4.1	25.8 ± 4.1	25.8 ± 3.9	0.01
Diabetes severity					
HbA1c,%	7.7 ± 1.6	7.8 ± 1.8	7.6 ± 1.5	7.6 ± 1.5	0.12
Diabetic duration, years	6.6 ± 5.7	6.8 ± 5.7	6.4 ± 5.7	6.7 ± 5.8	0.07
Diabetic duration grouping					0.04
<5 years	47.7	47.1	49.0	46.8	
5–10 years	21.1	20.9	19.7	23.1	
>10 years	31.2	32.0	31.2	30.0	
Diabetic neuropathy	33.3	36.0	32.5	30.8	0.05
Diabetic foot ulcer	3.9	4.2	3.4	4.3	0.11
Type of diabetes					0.10
Type 1	3.9	4.8	4.0	2.7	
Type 2	96.1	95.2	96.0	97.3	
Comorbidity					
Dyslipidemia	51.7	52.4	50.3	52.5	0.05
Hypertension	60.6	64.0	59.8	57.0	0.14
Ischemic heart disease	15.3	17.4	14.7	13.3	0.11
Chronic kidney disease	42.2	47.0	42.2	35.7	0.23
Stroke	6.4	8.0	6.2	4.6	0.13
Heart failure hospitalization	3.4	4.5	3.1	2.5	0.10
Myocardial infarction	3.4	3.6	3.6	2.8	0.05
Atrial fibrillation	2.8	3.2	2.1	3.2	0.07
Chronic obstructive pulmonary disease	8.1	8.9	6.8	9.0	0.08
Obstructive sleep apnea	4.6	4.4	3.7	6.1	0.12
Charlson’s Comorbidity Index score	3.6 ± 1.9	3.8 ± 2.0	3.6 ± 1.9	3.5 ± 1.7	0.12
Medication use					
Anti-platelet	23.7	25.4	22.0	23.8	0.08
Anti-coagulant	1.7	1.9	1.3	2.1	0.06
Statins	36.5	37.7	36.2	35.4	0.05
Fibrates	3.4	3.6	3.0	3.5	0.03
Ocular history					
Glaucoma	4.0	4.7	3.0	4.6	0.08
Age-related macular degeneration	26.8	23.4	25.2	33.8	0.24
Diabetic macular edema	43.3	39.7	45.3	45.4	0.12
Retinal vascular occlusion	6.7	6.5	7.1	6.6	0.03
Vitreous hemorrhage	20.3	25.0	18.7	16.0	0.22
Myopic choroidal neovascularization	1.9	1.3	2.2	2.2	0.07
All grade diabetic retinopathy	71.6	72.4	76.1	64.4	0.27
Received retinal laser	38.6	40.6	41.5	31.7	0.20
Received vitrectomy	6.3	6.5	6.5	5.6	0.04
No. of outpatient visits at ophthalmology in the previous year	3.9 ± 3.5	3.6 ± 3.5	4.1 ± 3.6	3.9 ± 3.3	0.14

Data were presented as frequency (percentage) or mean ± standard deviation. * Not included in the propensity score calculation. IPTW—inverse probability of treatment weighting; MASD—maximum absolute standardized difference; BMI—body mass index; HbA1C—glycated hemoglobin.

**Table 2:** Time to event outcome analysis in 6 months after inverse probability of treatment weighting.

	Incidence (95% CI) *			aHR (95%CI)/aSHR (95%CI)					
Outcome	Bevacizumab	Ranibizumab	Aflibercept	Bevacizumab vs. Ranibizumab	*p*	Bevacizumab vs. Aflibercept	*p*	Ranibizumab vs. Aflibercept	*p*
Major adverse cardiac event	1.99 (1.11–2.86)	2.76 (1.80–3.71)	1.23 (0.46–1.99)	0.52 (0.29–0.94)	0.032	0.96 (0.43–2.15)	0.914	1.83 (0.88–3.79)	0.106
Myocardial infarction	0.53 (0.07–0.99)	0.71 (0.22–1.20)	0.31 (−0.08–0.70)	0.41 (0.11–1.63)	0.208	0.58 (0.14–2.45)	0.460	1.41 (0.53–3.73)	0.495
Ischemic stroke	0.92 (0.32–1.53)	1.65 (0.91–2.40)	0.61 (0.06–1.16)	0.36 (0.16–0.82)	0.015	0.73 (0.22–2.45)	0.606	2.01 (0.75–5.38)	0.165
Cardiovascular death	0.86 (0.29–1.44)	0.44 (0.06–0.81)	0.34 (−0.06–0.73)	1.48 (0.47–4.66)	0.508	1.89 (0.41–8.80)	0.415	1.29 (0.26–6.31)	0.758
MALE outcome ^†^	2.95 (1.86–4.04)	2.00 (1.18–2.82)	0.74 (0.14–1.34)	1.21 (0.69–2.14)	0.504	3.24 (1.20–8.78)	0.021	2.67 (1.05–6.79)	0.039
Composite thromboembolic events ^‡^	2.16 (1.23–3.09)	2.53 (1.60–3.45)	1.48 (0.63–2.33)	0.65 (0.34–1.24)	0.190	0.84 (0.38–1.88)	0.678	1.30 (0.66–2.59)	0.450
Major bleeding	12.1 (9.8–14.3)	4.3 (3.1–5.5)	3.8 (2.4–5.1)	2.31 (1.63–3.27)	<0.001	2.27 (1.50–3.43)	<0.001	0.99 (0.62–1.56)	0.948
All-cause admission	58.6 (53.3–63.9)	27.6 (24.4–30.7)	30.2 (26.2–34.2)	1.76 (1.51–2.05)	<0.001	1.39 (1.16–1.65)	<0.001	0.79 (0.66–0.94)	0.007
All-cause death	3.26 (2.14–4.37)	0.55 (0.13–0.97)	2.61 (1.50–3.72)	5.53 (2.34–13.08)	<0.001	0.92 (0.51–1.67)	0.795	0.17 (0.07–0.41)	<0.001

Adjusted hazard ratio or adjusted sub-distribution hazard ratio were adjusted with variables with MASD more than 0.1 in Table 1, including age, diabetic foot, peripheral arterial disease, number of outpatient visits at ophthalmology in the previous year, hypertension, ischemic heart disease, chronic kidney disease, stroke, obstructive sleep apnea, Charlson’s comorbidity index score, sulfonylurea, insulin, cataract, retinal laser, age-related macular degeneration, diabetic macular edema, vitreous hemorrhage, all grade DR, HbA1c and creatinine; * Number of events per 100 person-years. ^†^ Composite of peripheral arterial disease, claudication, critical limb ischemia, percutaneous transluminal angioplasty, and amputation. ^‡^ Composite of myocardial infarction, ischemic stroke, transient ischemic attack, extremity thromboembolism and systemic thromboembolism. aHR—adjusted hazard ratio; aSHR—adjusted sub-distribution hazard ratio; CI—confidence interval; MALE—major adverse lower limb event.

## Data Availability

The data used for the current study cannot be made publicly available according to the NHIRD regulations on personal data protection, allowing only the person responsible for the data management to approach the data after approval from Taiwan NHI bureau.

## Supplementary Materials

The following supporting information can be downloaded at: https://www.mdpi.com/article/10.3390/jpm13030544/s1, Table S1: Baseline characteristics of patients after inverse probability of treatment weighting.
