# Supplementary material for: The Association of Intravitreal Injections of Different Anti-Vascular Endothelial Growth Factor with Systemic Outcomes in Diabetic Patients
Source: J Pers Med. 2023 Mar 18;13(3):544. doi: 10.3390/jpm13030544 (PMC10057023; doi:10.3390/jpm13030544)
Supplement: Supplementary file 1 [file jpm-13-00544-s001.zip › Supplemental Material.pdf]

Supplemental Table S1. Baseline characteristics of patients before inverse probability of treatment weighting.

Variable	Before IPTW Total (*n* = 3,040)	Bevacizumab (*n* = 1,477)	Ranibizumab (*n* = 1,056)	Aflibercept (*n* = 507)	MASD
Male	1,741 (57.3)	833 (56.4)	614 (58.1)	294 (58.0)	0.04
Age, years	61.8 ± 12.1	60.4 ± 13.0	62.5 ± 10.6	64.4 ± 11.7	**0.33**
Age ≥65 years	1,203 (39.6)	519 (35.1)	441 (41.8)	243 (47.9)	0.26
Number of injections during 6-month follow up	3.0 ± 2.2	1.7 ± 1.1	4.4 ± 2.4	3.8 ± 2.2	**1.46**
BMI, kg/m^2*	25.8 ± 4.1	25.8 ± 4.2	25.7 ± 4.1	25.8 ± 4.1	0.03
Diabetes severity					
HbA1c, %	7.8 ± 1.7	8.0 ± 2.0	7.6 ± 1.4	7.5 ± 1.4	**0.29**
Diabetic duration, years	6.6 ± 5.8	7.2 ± 5.8	6.0 ± 5.6	6.4 ± 6.0	**0.22**
Diabetic duration grouping					0.17
<5 years	1,467 (48.3)	652 (44.1)	554 (52.5)	261 (51.5)	
5-10 years	599 (19.7)	309 (20.9)	194 (18.4)	96 (18.9)	
>10 years	974 (32.0)	516 (34.9)	308 (29.2)	150 (29.6)	
Diabetic neuropathy	1,038 (34.1)	558 (37.8)	316 (29.9)	164 (32.3)	0.09
Diabetic foot	124 (4.1)	70 (4.7)	31 (2.9)	23 (4.5)	0.17
Type of diabetes					0.13
Type 1	139 (4.6)	84 (5.7)	40 (3.8)	15 (3.0)	
Type 2	2,901 (95.4)	1,393 (94.3)	1,016 (96.2)	492 (97.0)	
Comorbidity					
Dyslipidemia	1,558 (51.3)	818 (55.4)	488 (46.2)	252 (49.7)	0.18
Hypertension	1,888 (62.1)	1,019 (69.0)	585 (55.4)	284 (56.0)	**0.28**
Ischemic heart disease	502 (16.5)	282 (19.1)	144 (13.6)	76 (15.0)	0.15
Chronic kidney disease	1,360 (44.7)	770 (52.1)	415 (39.3)	175 (34.5)	**0.35**
Stroke	232 (7.6)	135 (9.1)	65 (6.2)	32 (6.3)	0.11
Heart failure hospitalization	126 (4.1)	81 (5.5)	29 (2.7)	16 (3.2)	0.14
Myocardial infarction	118 (3.9)	66 (4.5)	36 (3.4)	16 (3.2)	0.07
Atrial fibrillation	97 (3.2)	57 (3.9)	18 (1.7)	22 (4.3)	0.15

Variable	Before IPTW<br>Total<br>(*n* = 3,040)	Bevacizumab<br>(*n* = 1,477)	Ranibizumab<br>(*n* = 1,056)	Aflibercept<br>(*n* = 507)	MASD
Chronic obstructive pulmonary disease	241 (7.9)	123 (8.3)	65 (6.2)	53 (10.5)	0.16
Obstructive sleep apnea	131 (4.3)	66 (4.5)	30 (2.8)	35 (6.9)	**0.20**
Charlson's Comorbidity Index score	3.7 ± 2.0	3.9 ± 2.1	3.5 ± 1.9	3.6 ± 1.9	**0.21**
Medication use					
Anti-platelet	742 (24.4)	417 (28.2)	210 (19.9)	115 (22.7)	0.19
Anti-coagulant	63 (2.1)	33 (2.2)	12 (1.1)	18 (3.6)	0.17
Statins	1,103 (36.3)	578 (39.1)	354 (33.5)	171 (33.7)	0.12
Fibrates	107 (3.5)	62 (4.2)	30 (2.8)	15 (3.0)	0.07
Ocular history					
Glaucoma	132 (4.3)	75 (5.1)	34 (3.2)	23 (4.5)	0.09
Age-related macular degeneration	787 (25.9)	293 (19.8)	255 (24.1)	239 (47.1)	**0.62**
Diabetic macular edema	1,234 (40.6)	502 (34.0)	481 (45.5)	251 (49.5)	**0.32**
Retinal vascular occlusion	184 (6.1)	90 (6.1)	56 (5.3)	38 (7.5)	0.09
Vitreous hemorrhage	677 (22.3)	453 (30.7)	166 (15.7)	58 (11.4)	**0.46**
Myopic choroidal neovascularization	45 (1.5)	19 (1.3)	14 (1.3)	12 (2.4)	0.09
All grade diabetic retinopathy	2,257 (74.2)	1,057 (71.6)	876 (83.0)	324 (63.9)	**0.44**
Retinal laser	1,252 (41.2)	610 (41.3)	495 (46.9)	147 (29.0)	**0.36**
Vitrectomy	185 (6.1)	104 (7.0)	64 (6.1)	17 (3.4)	0.15
No. of outpatient visits at ophthalmology in the previous year	3.9 ± 3.6	3.1 ± 3.3	4.8 ± 3.8	4.3 ± 3.3	**0.47**

Abbreviations: IPTW, inverse probability of treatment weighting; MASD, maximum absolute standardized difference; BMI, body mass index; HbA1C, glycated hemoglobin.

Data were presented as frequency (percentage) or mean ± standard deviation.

* Not included in the propensity score calculation.
